# Tailoring Optical Gradient Force and Optical Scattering and Absorption Force

**DOI:** 10.1038/s41598-017-17874-1

**Published:** 2017-12-22

**Authors:** Junjie Du, Chi-Hong Yuen, Xiao Li, Kun Ding, Guiqiang Du, Zhifang Lin, C. T. Chan, Jack Ng

**Affiliations:** 1 Department of Physics, Hong Kong Baptist University, Hong Kong, China; 2 Quantum Institute for Light and Atoms, Department of Physics, East China Normal University, Shanghai, 200062 China; 3 Department of Physics and Institute for Advanced Studies, The Hong Kong University of Science and Technology, Hong Kong, China; 4 State Key Laboratory of Surface Physics, Key Laboratory of Micro and Nano Photonic Structures (MOE), and Department of Physics, Fudan University, Shanghai, China; 5 Institute of Computational and Theoretical Studies, Hong Kong Baptist University, Hong Kong, China; 6 Collaborative Innovation Center of Advanced Microstructures, Nanjing University, Nanjing, 210093 China

## Abstract

The introduction of the concept of gradient force and scattering and absorption force is an important milestone in optical trapping. However the profiles of these forces are usually unknown, even for standard setups. Here, we successfully calculated them analytically via multipole expansion and numerically via Mie theory and fast Fourier transform. The former provides physical insight, while the latter is highly accurate and efficient. A recipe to create truly conservative energy landscapes is presented. These may open up qualitatively new features in optical manipulation.

## Introduction

Optical force is an important tool to manipulate small particles. It has been fruitfully applied in a broad variety of areas, not only spanning the traditional scientific fields, but also in more applied fields^1–10^. It is customary and useful to theoretically split the optical force as **F** = **F**_**g**_ + **F**_**k**_, where **F**_g_ is the (conservative) gradient force and **F**_*k*_ is the (non-conservative) scattering and absorption force^1,11–19^. Here, ∇ × **F**_*g*_ = −∇ × ∇*U *= **0** and ∇⋅***F***_*k*_ = ∇⋅∇ × **g **= 0. By Stokes’ Theorem, ∇ × **F**_*g*_ = **0** indicates the work done by **F**_*g*_ is path independent and therefore a scalar potential energy *U* can be defined. By optical Earnshaw Theorem^11^, ∇⋅**F**_*k*_ = 0 indicates **F**_*k*_ alone cannot confine or trap a particle.

The concept of **F**_*g*_ and **F**_*k*_ are of paramount importance in optical manipulation. They have been guiding our intuition and interpretation^20^. However, their true profiles are not known in most situations, even for the most standard setup like optical tweezers (fundamental Gaussian beam). We remark that the formalism for calculating the total optical force induced by the standard optical tweezers are given in refs^21–24^, but **F**_*g*_ and **F**_*k*_ have not. It is highly desirable to calculate **F**_*g*_ and **F**_*k*_ independently. In principle, this could be achieved by using the Helmholtz Theorem^25^. However, calculation of **F**_*g*_ and **F**_*k*_ by Helmholtz Theorem at just a single location already requires two vector integrals over the entire open space, making it numerically impractical and physically non-transparent. In the literature, while the total optical force can be calculated^26,27^, people have not yet succeeded in separately calculating **F**_*g*_ and **F**_*k*_, except for the limiting cases of particle being small^11,14–16^ or large^13^ compare to the wavelength, but not the experimentally accessible micro-particles^11–13^. We note that there was previous attempt to compute the gradient force for Mie sized particle numerically^28^. However, our definition of gradient force is the conservative part of the total force, which is not what was calculated previously.

Small particles immersed in a fluid, such as colloids, will exhibit Brownian motion due to the random bombardment by the fluid molecules. If the particles are simultaneously illuminated by an intense laser, their motions can be strongly modified and controlled by optical forces. Tools like optical tweezers, arrays of optical traps^29–31^, or optical lattices^32^, are used extensively by researchers to produce “potential energy landscapes”^32–35^. While these approaches are very useful and the “potential energy landscapes” description does capture the physics, the potential energy is an effective one (the force is non-conservative, but can be considered as conservative because the particles are confined along the beam propagating direction). In the general case, one also need to bear in mind the non-conservative nature of optical forces^26,36–42^. We estimated that for a 1 micron radius polystyrene sphere trapped by a water immersion objective lens with N.A. = 1.3, on the focal plane, the maximum transverse non-conservative force amounts to about 5% of the maximum transverse conservative force. While this serves as a perturbation in many cases, we remark that the energy associated with the non-conservative force can accumulate while that of conservative force cannot. An example where the non-conservative force plays a significant role is a particle trapped by a circularly polarized Gaussian beam, where the non-conservative force would rotate the particle about the beam axis. Incident light flows in one direction, this induces non-conservative scattering and absorption force that cannot be described by a potential energy approach. The subtle distinction between conservative and non-conservative forces is that conservative/non-conservative forces can/cannot be derived from a potential energy and the associated work done is path independent/dependent. Consequently, they are of different attributes and have different applications. Generally speaking, ***F***_*g*_ and ***F***_*k*_ are, respectively, responsible for optical trapping^12^ and particle transportation^40^. They can also be combined to achieve other functionalities. For a conservative force, the textbook conservative classical mechanics and equilibrium statistical mechanics may be applied, which is in general significantly simpler than their non-conservative analog^43,44^. Furthermore, a colloid in a conservative force field can also be used to simulate other systems where experiments are more difficult^45,46^.

Here, we present an analytical and a numerical approach to calculate these forces. With these tools, we created a recipe to produce a fairly general class of conservative optical force field characterized by **F**_*k*_ = **0**. In general, particles immersed in an optical force field do not obey equilibrium statistical mechanics, making the analysis complicated^43,44^. With conservative forces, these issues are resolved. Such conservative optical force field is required in many applications of optical micromanipulation.

## Results

### Analytical Method

Based on a previously derived multipolar expression for optical forces acting on a spherical particle^19,36,47^, we derived in the supplemental material the analytical expression of **F**_*g*_ and **F**_*k*_ for the first few leading multipoles:

1 $$\begin{array}{rcl}{{\bf{F}}}_{g} & = & \frac{\alpha ^{\prime} }{4}\nabla {|{{\bf{E}}}_{in}|}^{2}+\frac{\beta ^{\prime} }{4}\nabla {|{{\bf{B}}}_{in}|}^{2}+\frac{\gamma ^{\prime} }{4}{\rm{Re}}\{\nabla \nabla {{{\bf{E}}}_{in}}^{\ast }\vdots (\nabla {{\bf{E}}}_{in}+\nabla {{\bf{E}}}_{in}^{T})\}\\  &  & +\frac{{\gamma ^{\prime} }_{m}}{4}{\rm{Re}}\{\nabla \nabla {{{\bf{B}}}_{in}}^{\ast }\vdots (\nabla {{\bf{B}}}_{in}+\nabla {{{\bf{B}}}_{in}}^{T})\}+\frac{{\rm{\Omega }}^{\prime} }{12}{\rm{Re}}\{\nabla \nabla \nabla {{{\bf{E}}}_{in}}^{\ast }\vdots sym(\nabla \nabla {{\bf{E}}}_{in})\}+O({k}^{9}{a}^{9}),\\ {{\bf{F}}}_{k} & = & -\frac{1}{2}\alpha ^{\prime\prime} \text{Im}\{\nabla {{\bf{E}}}_{in}^{\ast }\cdot {{\bf{E}}}_{in}\}-\frac{{k}^{4}}{12\pi {\varepsilon }_{0}c}\alpha ^{\prime} \beta ^{\prime} {\rm{Re}}\{{{\bf{E}}}_{in}\times {{\bf{B}}}_{in}^{\ast }\}\\  &  & -\frac{{k}^{5}}{40\pi {\varepsilon }_{0}}\alpha ^{\prime} \gamma ^{\prime} {\rm{Im}}\{(\nabla {{\bf{E}}}_{in}+\nabla {{\bf{E}}}_{in}^{T})\cdot {{\bf{E}}}_{in}^{\ast }\}+O({k}^{9}{a}^{9}),\end{array}$$

where *α′*, *β*′, *γ*′, *γ*′_*m*_, Ω′ and *α*″ are the multipole moments obtainable from Mie theory and are tabulated in the supplemental material, *k* is the wavenumber, *a* is the particle radius, and **E**_*in*_ and **B**_*in*_ are the arbitrary incident electromagnetic fields. In principle, one may keep adding higher order terms (such as octopole moment and beyond) into the multipole expansion to derive more accurate expression, but in practice the mathematics can be prohibitive, especially when Toroidal moments are involved. We note that Eq. (1) goes beyond the previous dipolar theory and reaches into the Mie regime.

### Numerical Method

Nevertheless, optically trapped particles often have sizes beyond the validity of Eq. (1). The real advantage of Eq. (1) lies in its transparent physics and insight, rather than its ability to compute the numerical values of **F**_*g*_ and **F**_*k*_. Here, we adopted an independent efficient numerical method based on fast Fourier transform (FFT) to treat particles with arbitrary sizes:

2 $$\begin{array}{rcl}{{\bf{F}}}_{g}({\bf{x}}) & = & \int \frac{{\bf{q}}[{\bf{q}}\cdot {\boldsymbol{F}}({\bf{q}})]/{q}^{2}}{{(2\pi )}^{3/2}}{e}^{i{\bf{q}}\cdot {\bf{x}}}{d}^{3}{\bf{q}},\\ {{\bf{F}}}_{k}({\bf{x}}) & = & \int \frac{[{\bf{q}}\times {\boldsymbol{F}}({\bf{q}})]\times {\bf{q}}/{q}^{2}}{{(2\pi )}^{3/2}}{e}^{i{\bf{q}}\cdot {\bf{x}}}{d}^{3}{\bf{q}},\end{array}$$

where **x** is the coordinate of the sphere center, ***F***(**q**) = (2*π*)^−3/2^∫**F**(**x**)*e* ^−*i****q***⋅***x***^ *d* ^3^ *x* is the Fourier transform of the total optical force **F**(**x**). We remark that the use of FFT in Eq. (2) significantly increases the computational speed and also the wavelet analysis can be an alternative to the FFT^48^. Eq. (2) can be applied to treat sufficiently fast decaying fields or periodic fields. The total optical force needed in the FFT is computed by

3 $${\bf{F}}({\bf{x}})={\oiint }_{{\rm{particle\; surface}}}\overleftrightarrow{{\bf{T}}}\cdot d{\bf{a}}$$

where

4 $$\overleftrightarrow{{\bf{T}}}=1/2\varepsilon {\bf{E}}{{\bf{E}}}^{\ast }+1/2\mu {\bf{H}}{{\bf{H}}}^{\ast }-1/4\varepsilon {|{\bf{E}}|}^{2}\overleftrightarrow{{\bf{I}}}-1/4\mu {|{\bf{H}}|}^{2}\overleftrightarrow{{\bf{I}}}$$

is the time averaged Maxwell stress tensor, with the required **E** and **H** calculated by the generalized Mie theory^22–24,26^, which at least within classical electrodynamics, is exact. It can be readily verified that in both Eqs (1) and (2), **F **= **F**_*g*_ + **F**_*k*_ while ∇ × **F**_*g*_ = **0** and ∇⋅**F**_*k*_ = 0, so they are indeed the gradient force and the scattering and absorption force.

**Figure 1:**
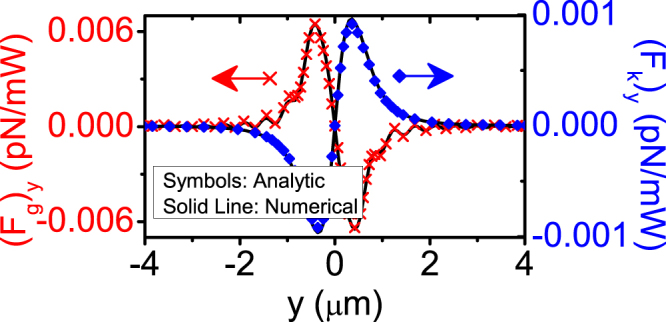
Remarkable agreement between analytical **(**Eq. (1)) and numerical **(**Eq. (2)) approaches is demonstrated. Gradient force (red) and scattering and absorption force (blue) calculated by the analytical expression Eq. (1) (symbols) and numerical approach Eq. (2) (solid lines), respectively. The 300 nm diameter particle of refractive index 1.59 is immersed in water (refractive index = 1.33). The wavelength is 1064 nm.

Figure 1 plotted **F**_*g*_ and **F**_*k*_ for a dielectric spherical particle with a diameter of 300 nm illuminated by an *x*-polarized fundamental Gaussian beam with a wavelength of 1064 nm, calculated analytically using Eq. (1) (symbols) and numerically using Eq. (2) (solid lines). The strongly focused Gaussian beam, which is known as optical tweezers, is modeled by using the highly accurate generalized vector Debye integral^31,49,50^, which is known to generate results that can be directly compared with experiments for particles larger than the wavelength. We remark that for particles smaller than wavelength, one must take into account the astigmatism to obtain an accurate result^51^. In short, the incident unfocused laser beam is illuminated on an objective lens with high numerical aperture. Since the lens is macroscopic in size, the focusing of light can be treated using geometrical optics with negligible errors. Then the vector Debye integral maps the geometrical optics solution to the field in the focal region. As far as the 300nm-diameter dielectric particle is concerned, excellent agreement is achieved. This validates both analytical and numerical approaches.

**Figure 2:**
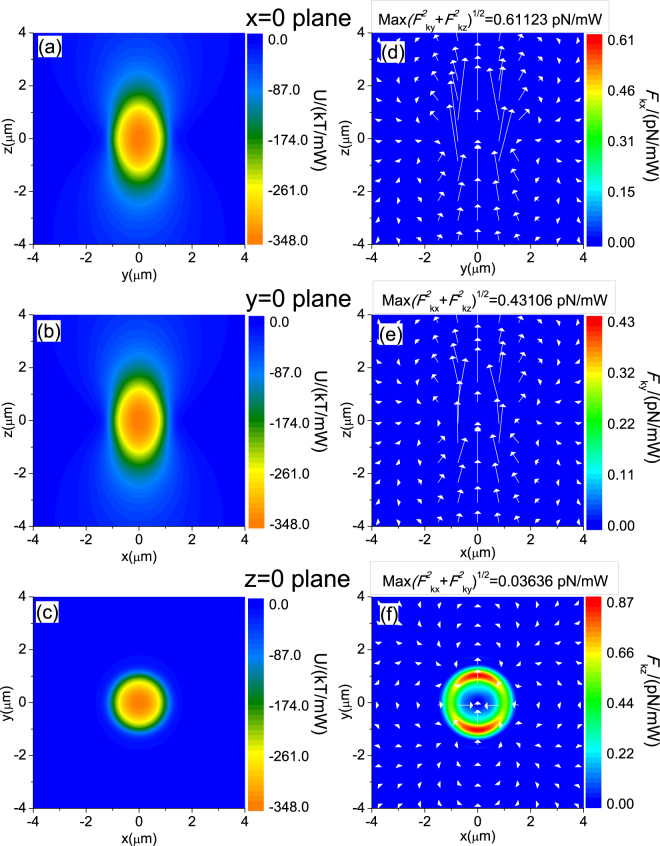
Fields pattern with a Gaussian beam illuminated on a particle. The incident beam is a strongly focused, *z*-propagating, and *x*-polarized fundamental Gaussian beam (i.e. optical tweezers) in water (*ε*_*water*_ = 1.33^2^). The Numerical Aperture N.A. is 1.3 and the filling factor is 1. Left: Potential energy *U* of the gradient force for a 2 micron diameter polystyrene particle, where **F**_*g*_ = −∇*U*. Right: Scattering and absorption force. Arrows indicate the direction and magnitude of force in logarithmic scale. Panels (a)-(b), (c)-(d), and (e)-(f) are for the *x* = 0, *y* = 0, and z = 0 planes, respectively.

In Fig. 2, we plotted **F**_*g*_ and **F**_*k*_ using the numerical approach (Eq. (2)), for the widely employed linearly polarized fundamental Gaussian beam. To obtain converged calculation for the strongly focused Gaussian beam, the unit cell for FFT is chosen such that the forces near the edges are at least two to three orders of magnitude smaller than that of the center. We also repeated our calculations with different unit cell sizes. For sufficiently large unit cell sizes, the results converge very well.

### Generation of a Conservative Force Field

**Figure 3:**
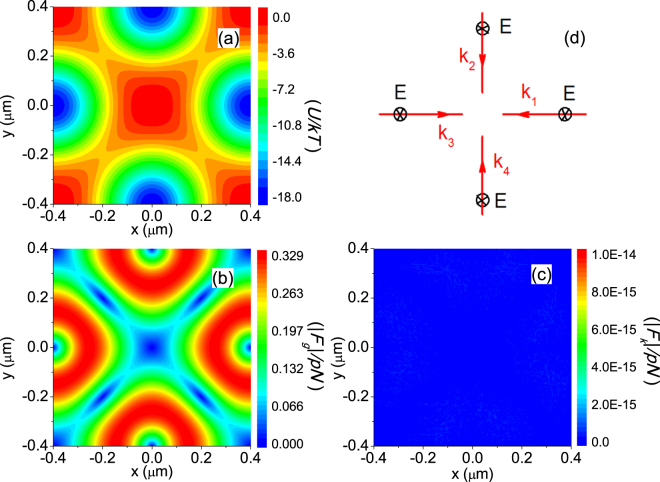
Conservative optical force acting on a 1 micron diameter particle. The particle in water is illuminated by a standing wave as depicted in panel **(**d**)**. (**a**) Potential Energy *U*. (**b**) |**F**_*g*_|. (**c**) |**F**_*k*_|. Clearly |**F**_*k*_| ≈ 0, therefore the force is conservative. (**d**) Schematic illustration for the configuration of the incident plane waves.

We now return to the generation of a conservative force field characterized by **F**_*k*_ = **0**. A careful inspection of Eq. (1) reveals that **F**_*k*_ = **0 **+ *O*(*ka*)^9^ if ${\rm{Im}}\{{{\bf{E}}}_{in}\}={\bf{0}}$, which is equivalent to having an incident standing wave. In other words, a standing wave can generate a conservative force field in three dimensions for particles with diameter less than roughly half a wavelength. Figure 3(a–c), plotted, respectively, the potential energy *U* (where **F**_*g*_ = −∇*U*), |**F**_*g*_|, and |**F**_*k*_| for a 1-micron diameter particle illuminated by a standing wave generated by interfering four plane waves (each with an intensity of 10^4^ W/cm^2^). Clearly, a conservative periodic potential is generated, since |**F**_*k*_| in Fig. 3(c) is very small, on the same order as the numerical noise. We note that a 1-micron diameter particle is beyond the validity of Eq. (1), and therefore the observed conservative force is not a prediction of Eq. (1). We repeated the calculation with 5 microns diameter particle (data not shown), still **F**_*k*_ is comparable with the numerical noise. These findings are consequences of a more general theorem we analytically proved in the supplemental material: for a spherical particle illuminated by TE or TM standing wave in two dimensions (i.e. all incident wave vectors lie on the *z* = 0 plane), the optical force is conservative, just as shown in Fig. 3. We remark that the vanishing of the scattering and absorption force is a consequence of symmetry (see supplemental material). We can generate a conservative force even for a lossy, absorptive particle (data not shown), according to our analytical proof in the supplemental material.

**Figure 4:**
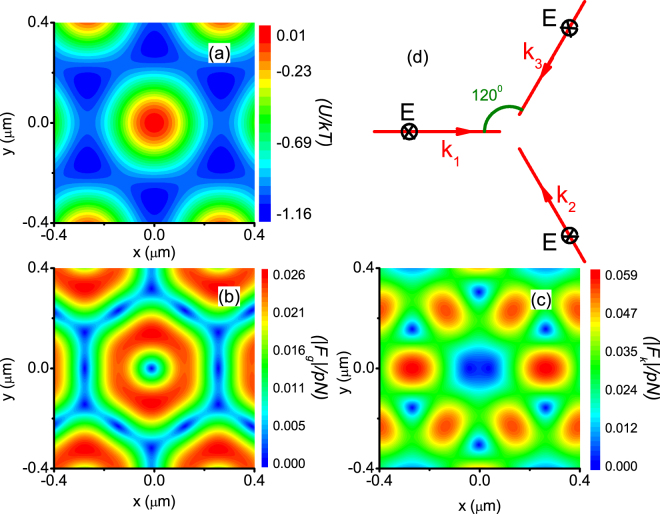
Nonconservative optical force when the incident wave is not a standing wave. (**a**) Potential Energy *U*. (**b**) |**F**_*g*_|. (**c**) |**F**_*k*_|. (**d**) Schematic illustration for the configuration of the incident plane waves.

For comparison, the forces acting on a 1-micron diameter particle when illuminated by three plane waves are plotted in Fig. 4. These three plane waves do not form a standing wave, but their incident momentum do cancel each other completely, i.e. they have the same amplitude and $\sum _{i=1}^{3}{{\bf{k}}}_{i}={\bf{0}}$. When the waves are coherent, both **F**_*k*_ and **F**_*g*_ are non-zero due to interference. The force field is clearly non-conservative, as the maximum value of **F**_*k*_ is actually greater than that of **F**_*g*_. This highlights the importance of having a standing wave.

## Discussion

In summary, we devised an analytical and a numerical approach to calculate the gradient force and the scattering and absorption force for the experimentally accessible micro-particles. The profile of these forces associated with the widely employed optical tweezers is presented. This will enable more detailed analysis and precise control on optical micromanipulation. As an example, we provided a recipe and a sufficient condition to generate or tailor a conservative force field for particles smaller than half a wavelength using arbitrary standing wave. For TE or TM incident standing wave in two dimensions, the induced force field is conservative for spherical particle of any size. This will allow us to create a truly conservative force field, paving the way to mimic a wide variety of phenomena in equilibrium statistical mechanics using optical micromanipulation system. Finally, we remark that our approach can also be used in other non-conservative force field, such as the acoustic force.

## Electronic supplementary material

Supplementary information
