# Supplementary material for: Tailoring Optical Gradient Force and Optical Scattering and Absorption Force
Source: Sci Rep. 2017 Dec 22;7:18042. doi: 10.1038/s41598-017-17874-1 (PMC5741730; doi:10.1038/s41598-017-17874-1)
Supplement: Supplementary file 1 — Supplementary information [file 41598_2017_17874_MOESM1_ESM.docx]

**Supplemental Materials –Tailoring Optical Gradient Force and Optical Scattering and Absorption Force**

Junjie Du,^1,2†^ Chi-Hong Yuen,^1†^ Xiao Li,^1^ Kun Ding,^3^ Guiqiang Du,^1^
Zhifang Lin,^4^ C. T. Chan,^3^ & Jack Ng^1,5*^

^1^Department of Physics, Hong Kong Baptist University, Hong Kong, China

^2^Quantum Institute for Light and Atoms, Department of Physics, East China Normal University, Shanghai 200062, China

^3^Department of Physics and Institute for Advanced Studies, The Hong Kong University of Science and Technology, Hong Kong, China

^4^State Key Laboratory of Surface Physics, Key Laboratory of Micro and Nano Photonic Structures (MOE), and Department of Physics, Fudan University, Shanghai, China

^5^Institute of Computational and Theoretical Studies, Hong Kong Baptist University, Hong Kong, China

^*^ jacktfng@hkbu.edu.hk

^†^these authors contributed equally to this work

**A1. Analytically splitting the optical force into gradient force and scattering & absorption force for small to intermediately sized particles**

According to Helmholtz theorem [[[1]](#endnote-1)], the optical force field in three dimensions (and also any other vector fields) can be decomposed into

$$\mathbf{F}=\mathbf{F}_{\mathbf{g}}+\mathbf{F}_{\mathbf{k}},$$

where

and and $\mathbf{g}$ are arbitrary functions in 3D. The curl-less $\mathbf{F}_{\mathbf{g}}$ is the gradient force and the divergence-less $\mathbf{F}_{\mathbf{k}}$ is the scattering & absorption force.

Here, we consider a homogeneous and isotropic sphere. The analytical multipolar expression for optical force is tabulated in Eq. (32) of ref. [[2]](#endnote-2). By truncating terms on the order of$\left(ka\right)^{9}$ or smaller, one arrives at

$\mathbf{F}=\mathbf{F}_{p}+\mathbf{F}_{m}+\mathbf{F}_{Qe}+\mathbf{F}_{Qm}+\mathbf{F}_{\Omega e}+\mathbf{F}_{p-m}+\mathbf{F}_{Qe-p}+O\left(ka\right)^{9}$,

where

$$\mathbf{F}_{p}=\frac{1}{2}\mathrm{Re}\{(\nabla\mathbf{E}_{in}^{*})\cdot\mathbf{p}\}$$

is the interception force due to electric dipole ($\mathbf{p}$),

$$\mathbf{F}_{m}=\frac{1}{2}\mathrm{Re}\{(\nabla\mathbf{B}_{in}^{*})\cdot\mathbf{m}\}$$

is the interception force due to the magnetic dipole ($\mathbf{m}$),

$$\mathbf{F}_{Qe}=\frac{1}{4}\mathrm{Re}\{(\nabla\nabla\mathbf{E}_{in}^{*}):\overleftrightarrow{\mathbf{Q}}_{e})\}$$

is the interception force due to the electric quadrupole ($\overleftrightarrow{\mathbf{Q}}_{e}$),

$$\mathbf{F}_{Qm}=\frac{1}{4}\mathrm{Re}\{(\nabla\nabla\mathbf{B}_{in}^{*}):\overleftrightarrow{\mathbf{Q}}_{m})\}$$

is the interception force due to the magnetic quadrupole ($\overleftrightarrow{\mathbf{Q}}_{m}$),

$$\mathbf{F}_{\Omega e}=\frac{1}{12}\mathrm{Re}\{(\nabla\nabla\nabla\mathbf{E}_{in}^{*})\vdots\overleftrightarrow{\mathbf{\Omega}}_{e})\}$$

is the interception force due to the electric octopole ($\overleftrightarrow{\mathbf{\Omega}}_{e}$),

$$\mathbf{F}_{p-m}=-\frac{k^{4}}{12\pi\varepsilon_{0}c}\mathrm{Re}\{\mathbf{p}\times\mathbf{m}^{*}\}$$

is the recoil force due to the interference between the electric dipole and magnetic dipole, and

$$\mathbf{F}_{Qe-p}=-\frac{k^{5}}{40\pi\varepsilon_{0}}\mathrm{Im}\{\overleftrightarrow{\mathbf{Q}}_{e}\cdot\mathbf{p}*\}$$

is the recoil force due to the interference between the electric dipole and electric quadrupole. The multipole moments can be found by the Mie scattering theory [[[3]](#endnote-3)]:

,

,

,

,

,

with $\alpha'$,$\beta'$,$\gamma'$,$\eta'$,$\zeta'$and $\alpha''$,$\beta''$,$\gamma''$,$\eta''$,$\zeta''$ respectively denote the real and imaginary parts of the polarizabilities,

$$\begin{matrix} a_{n}=\frac{m\psi_{n}(mka)\psi_{n}^{'}(ka)-\psi_{n}(ka)\psi_{n}^{'}(mka)}{m\psi_{n}(mka)\xi_{n}^{'}(ka)-\xi_{n}(ka)\psi_{n}^{'}(mka)} \\ b_{n}=\frac{\psi_{n}(mka)\psi_{n}^{'}(ka)-m\psi_{n}(ka)\psi_{n}^{'}(mka)}{\psi_{n}(mka)\xi_{n}^{'}(ka)-m\xi_{n}(ka)\psi_{n}^{'}(mka)} \end{matrix}$$

are the standard Mie coefficients [3], $m$is the relative refractive index, *k* is the wave number, *a* is the particle radius, and $\psi_{n}$ and $\xi_{n}$ are the Riccati-Bessel functions. From the Mie coefficients, the leading orders of the multipole moments in *ka* can be derived: $\alpha'\sim(ka)^{3}$, $\beta'\sim(ka)^{5}$,$\gamma'\sim(ka)^{5}$,$\alpha''\sim(ka)^{6}$,$\eta'\sim(ka)^{7}$,$\zeta'\sim(ka)^{7}$, $\beta''\sim(ka)^{10}$ $\gamma''\sim(ka)^{10}$,$\eta''\sim(ka)^{14}$and $\zeta''\sim(ka)^{14}$. Clearly, terms with $\beta''$, $\gamma''$, $\eta''$, and $\zeta''$ or smaller can be neglected. Here $\mathbf{E}_{in}$ and $\mathbf{B}_{in}$ are the incident electromagnetic field. Accordingly, once we know the multipole moments, we can compute the optical force without explicitly solving the scattering problem. For this reason, the formalism is considered to be analytical.

We shall now split the optical force into $\mathbf{F}_{g}$ and $\mathbf{F}_{\mathbf{k}}$.The electric dipole term has been treated by Ashkin [[[4]](#endnote-4)]:

where the first term is part of the gradient force (since it is curl-less) and the second term is part of the scattering and absorption force (since it is divergence-less).

Similarly, $\mathbf{F}_{m}$can be treated using a parallel procedure [[[5]](#endnote-5)]:

therefore it belongs to the gradient force. For the quadrupole term, by using , one arrives at

$$\begin{matrix} \mathbf{F}_{Qe}=\mathrm{Re}\left\{\nabla\nabla\mathbf{E}_{in}^{*}:\overleftrightarrow{\mathbf{Q}}_{e}\right\} \\ =\mathrm{Re}\left\{\nabla\nabla_{i}\left(\mathbf{E}_{in}\right)_{j}^{*}:\gamma\left[\nabla_{j}\left(\mathbf{E}_{in}\right)_{i}+\nabla_{i}\left(\mathbf{E}_{in}\right)_{j}\right]\right\} \\ =\left(\gamma'/2\right)\nabla\left|\nabla_{i}\left(\mathbf{E}_{in}\right)_{j}\right|^{2}+\left(\gamma'/2\right)\nabla\mathrm{Re}\left\{\nabla_{i}\left(\mathbf{E}_{in}\right)_{j}^{*}:\nabla_{j}\left(\mathbf{E}_{in}\right)_{i}\right\}+O(ka)^{9}, \end{matrix}$$

which is clearly derivable from a gradient of a potential and thus it is part of the gradient force.

Similarly, by using , one has

$$\mathbf{F}_{Qm}=\left(\eta'/2\right)\nabla\left|\nabla_{i}\left(\mathbf{B}_{in}\right)_{j}\right|^{2}+\left(\eta'/2\right)\nabla\mathrm{Re}\left\{\nabla_{i}\left(\mathbf{B}_{in}\right)_{j}^{*}:\nabla_{j}\left(\mathbf{B}_{in}\right)_{i}\right\}+O(ka)^{9},$$

therefore it is also part of the gradient force.

For$\mathbf{F}_{\Omega e}$, by using , one has

$$\begin{matrix} \mathbf{F}_{\Omega e}=\mathrm{Re}\{(\nabla\nabla\nabla\mathbf{E}_{in}^{*})\vdots\zeta sym(\nabla\nabla\mathbf{E}_{in})\} \\ =\left(1/6\right)\mathrm{Re}\{\zeta\{\nabla[sym(\nabla_{i}\nabla_{j}\left(\mathbf{E}_{in}\right)_{k}^{*})sym(\nabla_{k}\nabla_{j}\left(\mathbf{E}_{in}\right)_{i})]\}\} \\ =\left(\zeta'/12\right)\nabla\left|sym(\nabla\nabla\mathbf{E}_{in})\right|^{2}+O(ka)^{9} \end{matrix}$$

where $sym(\nabla\nabla\mathbf{E}_{in})$denotes the sum of all permutations subtracted by its trace. Doubtlessly, this is part of the gradient force.

Regarding $\mathbf{F}_{p-m}$,

$$\begin{matrix} \mathrm{Re}\{\mathbf{p}\times\mathbf{m}^{*}\}=\mathrm{Re}\{\alpha\beta^{*}(\mathbf{E}_{in}\times\mathbf{B}^{*}_{in})\} \\ =\alpha'\beta'\mathrm{Re}(\mathbf{E}_{in}\times\mathbf{B}^{*}_{in})+O(ka)^{9}. \end{matrix}$$

Taking the divergence of , one arrives at

$$\nabla\cdot\{\alpha'\beta'\mathrm{Re}[\mathbf{E}_{in}\times\mathbf{B}_{in}^{*}]\}=\alpha'\beta'\mu_{0}(\nabla\cdot<\mathbf{S}_{in}>)=0$$

Therefore it belongs to the scattering force.

For the last term$\mathbf{F}_{Qe-p}$

$$\begin{matrix} \mathrm{Im}\{\overleftrightarrow{\mathbf{Q}}_{e}\cdot\mathbf{p}^{*}\}=\mathrm{Im}\{\gamma(\nabla\mathbf{E}_{in}+\nabla\mathbf{E}_{in}^{T})\cdot\alpha\mathbf{E}_{in}^{*}\} \\ =\gamma'\alpha'\mathrm{Im}\{[\nabla_{i}E_{j}+\nabla_{j}E_{i}]E_{j}^{*}\}+O(ka)^{9} \end{matrix}$$

Taking divergence of , one arrives at

$$\begin{matrix} \nabla\cdot\gamma'\alpha'\mathrm{Im}\left\{E_{j}^{*}\nabla_{i}E_{j}+E_{j}^{*}\nabla_{j}E_{i}\right\} \\ =\gamma'\alpha'\mathrm{Im}\left\{\nabla_{i}\left(E_{j}^{*}\nabla_{i}E_{j}\right)\right\}+\gamma'\alpha'\mathrm{Im}\left\{\nabla_{i}\left(E_{j}^{*}\nabla_{j}E_{i}\right)\right\} \\ =\gamma'\alpha'\mathrm{Im}\left\{\left(E_{j}^{*}\nabla^{2}E_{j}\right)\right\}+\gamma'\alpha'\mathrm{Im}\left\{\left(\nabla_{j}E_{i}\right)\nabla_{i}E_{j}^{*}\right\} \\ =\gamma'\alpha'\mathrm{Im}\left\{\left(-k^{2}\left|E\right|^{2}\right)\right\}+\left(\gamma'\alpha'/2\right)\mathrm{Im}\left\{\left(\nabla_{j}E_{i}\right)\nabla_{i}E_{j}^{*}+\left(\nabla_{i}E_{j}\right)\nabla_{j}E_{i}^{*}\right\} \\ =\left(\gamma'\alpha'/2\right)\mathrm{Im}\{\mathrm{Re}\left[\left(\nabla_{j}E_{i}\right)\nabla_{i}E_{j}^{*}\right]\} \\ =0 \end{matrix}$$

Hence it must be part of the scattering force.

Combining (3) to (9), we finally arrives at the gradient force

$$\begin{matrix} \mathbf{F}_{g}=\frac{1}{4}\alpha'(\nabla|\mathbf{E}_{in}|^{2})+\frac{1}{4}\beta'(\nabla|\mathbf{B}_{in}|^{2})+\frac{1}{4}\gamma'\mathrm{Re}\{(\nabla\nabla\mathbf{E}_{in}^{*}):(\nabla\mathbf{E}_{in}+\nabla\mathbf{E}_{in}^{T})\} \\ +\frac{1}{4}\eta'\mathrm{Re}\{(\nabla\nabla\mathbf{B}_{in}^{*}):(\nabla\mathbf{B}_{in}+\nabla\mathbf{B}_{in}^{T}))\}+\frac{1}{12}\zeta'\mathrm{Re}\{(\nabla\nabla\nabla\mathbf{E}_{in}^{*})\vdots sym(\nabla\nabla\mathbf{E}_{in})\}+O(ka)^{9} \end{matrix}$$

and the scattering and absorption force

$$\begin{matrix} \mathbf{F}_{k}=\frac{1}{2}\alpha''\mathrm{Im}\{\nabla\mathbf{E}_{in}^{*}\cdot\mathbf{E}_{in}\}-\frac{k^{4}}{12\pi\varepsilon_{0}c}\mathrm{Re}\{\mathbf{p}\times\mathbf{m}^{*}\} \\ -\frac{k^{5}}{40\pi\varepsilon_{0}}\gamma'\alpha'\mathrm{Im}[(\nabla\mathbf{E}_{in}+\nabla\mathbf{E}_{in}^{T})\cdot\mathbf{E}_{in}]+O(ka)^{9}. \end{matrix}$$

Generally speaking, the expression and are quantitatively correct for particle with size less than 40% of a wavelength.

**A2. 2D TE standing wave produces a conservative optical force for spherical particle**

An incident electromagnetic standing wave is a non-propagating wave characterized by a vanishing Poynting vector everywhere:

$$\mathrm{Re}\left\{\mathbf{S}_{in}\right\}=\mathrm{Re}\left\{\mathbf{E}_{in}\times\mathbf{H}_{in}^{*}\right\}=\mathbf{0}.$$

Without loss of generality and up to an insignificant constant phase factor in $\mathbf{E}_{in}$, the condition can be rephrased as

$\mathrm{Im}\left\{\mathbf{E}_{in}\right\}=\mathbf{0}$.

Consider a particle located at position $\mathbf{a}$, the TE incident field can be decomposed into a series of *z*-polarized plane wave:

$$\mathbf{E}_{in}=\int E_{0\mathbf{k}}e^{i\mathbf{k}\cdot\mathbf{a}+i\delta_{\mathbf{k}}}\hat{\mathbf{z}}e^{i\mathbf{k}\cdot\mathbf{r}}d\Omega_{k},$$

where $E_{0\mathbf{k}}=E_{0,-\mathbf{k}}$ and are the amplitude and phase of the incident plane wave characterized by wave vector$\mathbf{k}$. The integration is over all $\mathbf{k}$ that fulfills

$$\begin{matrix} k_{z}=0, \\ \left|\mathbf{k}\right|=\omega/c, \end{matrix}$$

where $\omega$is the frequency of incident light, $c$ is the speed of light, and $\mathbf{r}$ is the position vector measured from the particle position $\mathbf{a}$.

According to Ref. 2, the total time averaged optical force is

$\mathbf{F}=\mathbf{F}_{\text{intercept}}+\mathbf{F}_{\text{recoil}}$,

where the interception force $\mathbf{F}_{\text{intercept}}$ and the recoil force $\mathbf{F}_{\text{recoil}}$ are respectively denoted by $\left\langle\vec{\mathbf{F}}_{mix}\right\rangle$ and $\left\langle\vec{\mathbf{F}}_{s}\right\rangle$ in the Supplementary Information of ref. 2. Microscopically, light scattering by a small particle consists of two separate processes. In the first process, the photons are absorbed and this leads to a momentum transfer given by $\mathbf{F}_{\text{intercept}}$. In the second process, some of the absorbed photons will be re-emitted, leading to a recoil force given by$\mathbf{F}_{\text{recoil}}$.

**A2a. The conservativeness of the interception force**

We define $\mathbf{f}(\mathbf{k},\mathbf{k}')$ as the scattering amplitude for the particle illuminated by $\hat{\mathbf{z}}e^{i\mathbf{k}'\cdot\mathbf{r}}$. For the incident field , the full scattering amplitude is given by the superposition of all $\mathbf{f}(\mathbf{k},\mathbf{k}')$with the corresponding weighing:

$\mathbf{F}(\mathbf{k},\mathbf{a})=\oint\mathbf{f}(\mathbf{k},\mathbf{k}')E_{0k'}e^{i(\mathbf{k}'\cdot\mathbf{a}+\delta_{k'})}d\Omega_{k'}$.

The interception force is given by (see Eq. 16 of ref. 2)

$\mathbf{F}_{\text{intercept}}=-\frac{1}{c}\oint_{S_{\infty}} \left\langle\mathbf{S}_{mix}\right\rangle d\sigma$,

where $S_{\infty}$is a sphere with an infinite radius,

.

By using Jones’ lemma (see Eq. (8)-(11) of the Supplementary Information of ref. 2 ), we arrive at

$$\mathbf{F}_{\text{intercept}}=2\pi\sqrt{\mu_{0}/\varepsilon_{0}}/k\oint\mathrm{Re}\left\{iE_{0\mathbf{k}}e^{-i\mathbf{k}\cdot\mathbf{a}-i\delta_{\mathbf{k}}}F_{z}\left(\mathbf{k},\mathbf{a}\right)\right\}\hat{\mathbf{k}}d\Omega_{k}$$

Splitting the integral into the $+\mathbf{k}$ and $-\mathbf{k}$ components:

$$\mathbf{F}_{\text{intercept}}=\left(2\pi\sqrt{\mu_{0}/\varepsilon_{0}}/k\right)\int\mathrm{Re}\left\{iE_{0\mathbf{k}}e^{-i\mathbf{k}\cdot\mathbf{a}-i\delta_{\mathbf{k}}}F_{z}\left(\mathbf{k},\mathbf{a}\right)-iE_{0\mathbf{k}}e^{i\mathbf{k}\cdot\mathbf{a}+i\delta_{\mathbf{k}}}F_{z}\left(-\mathbf{k},\mathbf{a}\right)\right\}\hat{\mathbf{k}}d\Omega_{k}$$

Using , one arrives at

$\mathbf{F}_{\text{intercept}}=2\pi\sqrt{\mu_{0}/\varepsilon_{0}}/k\int\left\{\mathrm{Re}\left\{i\left[\begin{matrix} +E_{0\mathbf{k}}E_{0\mathbf{k}'}\int f_{z}(+\mathbf{k},\mathbf{k}')e^{i(\mathbf{k}'-\mathbf{k})\cdot\mathbf{a}+i(\delta_{k'}-\delta_{k})}d\Omega_{k'}\hat{\mathbf{k}} \\ -E_{0\mathbf{k}}E_{0\mathbf{k}'}\int f_{z}(-\mathbf{k},\mathbf{k}')e^{i(\mathbf{k}'+\mathbf{k})\cdot\mathbf{a}+i(\delta_{k'}+\delta_{k}))}d\Omega_{k'}\hat{\mathbf{k}} \end{matrix}\right]\right\}\right\}d\Omega_{k}$.

Using the change of variable $\mathbf{k}'\to-\mathbf{k}'$ for the second term in , one has

$\mathbf{F}_{\text{intercept}}=2\pi\sqrt{\mu_{0}/\varepsilon_{0}}/k\int\left\{\mathrm{Re}\left\{i\left[\begin{matrix} +E_{0\mathbf{k}}E_{0\mathbf{k}'}\int f_{z}(+\mathbf{k},\mathbf{k}')e^{i((\mathbf{k}'-\mathbf{k})\cdot\mathbf{a}+(\delta_{k'}-\delta_{k})}d\Omega_{k'}\hat{\mathbf{k}} \\ -E_{0\mathbf{k}}E_{0\mathbf{k}'}\int f_{z}(-\mathbf{k},-\mathbf{k}')e^{i((-\mathbf{k}'+\mathbf{k})\cdot\mathbf{a}+(-\delta_{k'}+\delta_{k}))}d\Omega_{k'}\hat{\mathbf{k}} \end{matrix}\right]\right\}\right\}d\Omega_{k}$.

We note that all wave vectors $\mathbf{k}$ are lying on the *xy*-plane, therefore by rotating the system along the *z*-axis by 180^o^, we have $f_{z}(\mathbf{k},\mathbf{k}')=f_{z}(-\mathbf{k},-\mathbf{k}')$. Therefore Eq. becomes

$$\begin{matrix} \mathbf{F}_{\text{intercept}}=2\pi\sqrt{\mu_{0}/\varepsilon_{0}}/k\int\left\{\mathrm{Re}\left\{i\left[\begin{matrix} +E_{0\mathbf{k}}E_{0\mathbf{k}'}\int f_{z}(\mathbf{k},\mathbf{k}')e^{i((\mathbf{k}'-\mathbf{k})\cdot\mathbf{a}+(\delta_{k'}-\delta_{k})}d\Omega_{k'}\hat{\mathbf{k}} \\ -E_{0\mathbf{k}}E_{0\mathbf{k}'}\int f_{z}(\mathbf{k},\mathbf{k}')e^{i((-\mathbf{k}'+\mathbf{k})\cdot\mathbf{a}+(-\delta_{k'}+\delta_{k}))}d\Omega_{k'}\hat{\mathbf{k}} \end{matrix}\right]\right\}\right\}d\Omega_{k} \\ =2\pi\sqrt{\mu_{0}/\varepsilon_{0}}/k\int\left\{\mathrm{Re}\left\{iE_{0\mathbf{k}}E_{0\mathbf{k}'}\oint f_{z}(\mathbf{k},\mathbf{k}')\left[c(\mathbf{k}',\mathbf{k})-c(\mathbf{k},\mathbf{k}')\right]d\Omega_{k'}]\hat{\mathbf{k}}\right\}\right\}d\Omega_{k} \end{matrix}$$

where

$c(\mathbf{k}',\mathbf{k})=e^{i(\mathbf{k}'-\mathbf{k})\cdot\mathbf{a}+i(\delta_{k'}-\delta_{k})}$.

Now, we can prove $\mathbf{F}_{\text{intercept}}$ is conservative by showing that$\nabla_{\mathbf{a}}\times\mathbf{F}_{\text{intercept}}=\mathbf{0}$ (where operates on the particle position **a**):

$$\begin{matrix} \nabla_{\mathbf{a}}\times\mathbf{F}_{\text{intercept}}=2\pi\sqrt{\mu_{0}/\varepsilon_{0}}/k\nabla_{\mathbf{a}}\times\int\left\{\mathrm{Re}\left\{iE_{0\mathbf{k}}E_{0\mathbf{k}'}\oint f_{z}(\mathbf{k},\mathbf{k}')\left[c(\mathbf{k}',\mathbf{k})-c(\mathbf{k},\mathbf{k}')\right]d\Omega_{k'}\hat{\mathbf{k}}\right\}\right\}d\Omega_{k} \\ =2\pi\sqrt{\mu_{0}/\varepsilon_{0}}/k\int\left\{\mathrm{Re}\left\{iE_{0\mathbf{k}}E_{0\mathbf{k}'}\nabla_{\mathbf{a}}\left(\oint f_{z}(\mathbf{k},\mathbf{k}')\left[c(\mathbf{k}',\mathbf{k})-c(\mathbf{k},\mathbf{k}')\right]d\Omega_{k'}\right)\times\hat{\mathbf{k}}\right\}\right\}d\Omega_{k} \\ =2\pi\sqrt{\mu_{0}/\varepsilon_{0}}/k\int\left\{\mathrm{Re}\left\{iE_{0\mathbf{k}}E_{0\mathbf{k}'}\left(\oint f_{z}(\mathbf{k},\mathbf{k}')i(\mathbf{k}'-\mathbf{k})\left[c(\mathbf{k}',\mathbf{k})+c(\mathbf{k},\mathbf{k}')\right]d\Omega_{k'}\right)\times\hat{\mathbf{k}}\right\}\right\}d\Omega_{k} \\ =2\pi\sqrt{\mu_{0}/\varepsilon_{0}}/k\int\left\{\mathrm{Re}\left\{iE_{0\mathbf{k}}E_{0\mathbf{k}'}\left(\oint f_{z}(\mathbf{k},\mathbf{k}')i(\mathbf{k}'\times\hat{\mathbf{k}})\left[c(\mathbf{k}',\mathbf{k})+c(\mathbf{k},\mathbf{k}')\right]d\Omega_{k'}\right)\right\}\right\}d\Omega_{k} \end{matrix}$$

Interchanging $\mathbf{k}$and $\mathbf{k}'$ in :

$$\begin{matrix} \nabla_{\mathbf{a}}\times\mathbf{F}_{\text{intercept}}=2\pi\sqrt{\mu_{0}/\varepsilon_{0}}/k\int\left\{\mathrm{Re}\left\{iE_{0\mathbf{k}'}E_{0\mathbf{k}}\left(\oint f_{z}(\mathbf{k}',\mathbf{k})i(\hat{\mathbf{k}}\times\mathbf{k}')\left[c(\mathbf{k},\mathbf{k}')+c(\mathbf{k}',\mathbf{k})\right]d\Omega_{k}\right)\right\}\right\}d\Omega_{k'} \\ =2\pi\sqrt{\mu_{0}/\varepsilon_{0}}/k\int\left\{\mathrm{Re}\left\{iE_{0\mathbf{k}'}E_{0\mathbf{k}}\left(\oint f_{z}(\mathbf{k},\mathbf{k}')i(\hat{\mathbf{k}}\times\mathbf{k}')\left[c(\mathbf{k},\mathbf{k}')+c(\mathbf{k}',\mathbf{k})\right]d\Omega_{k'}\right)\right\}\right\}d\Omega_{k} \\ =-2\pi\sqrt{\mu_{0}/\varepsilon_{0}}/k\int\left\{\mathrm{Re}\left\{iE_{0\mathbf{k}'}E_{0\mathbf{k}}\left(\oint f_{z}(\mathbf{k},\mathbf{k}')i(\mathbf{k}'\times\hat{\mathbf{k}})\left[c(\mathbf{k},\mathbf{k}')+c(\mathbf{k}',\mathbf{k})\right]d\Omega_{k'}\right)\right\}\right\}d\Omega_{k} \\ =-\nabla_{\mathbf{a}}\times\mathbf{F}_{\text{intercept}} \end{matrix}$$

where we have used the reciprocity condition:

$f_{z}(\mathbf{k},\mathbf{k}')=f_{z}(\mathbf{k}',\mathbf{k})$,

and the last line of follows from . Accordingly

$\nabla_{\mathbf{a}}\times\mathbf{F}_{\text{intercept}}=\mathbf{0}$.

Therefore $\mathbf{F}_{\text{intercept}}$is curl-less, and it is part of the gradient force.

**A2b. The conservativeness of the recoil force**

Consider a general incident transverse electric (TE) standing wave, which consists of *N* pairs of counter-propagating plane waves satisfying and .

$\mathbf{E}_{in}(\mathbf{r}+\mathbf{a})=\mathbf{\varepsilon}_{\mathbf{k}}e^{i\mathbf{k}\cdot\mathbf{r}}=\sum_{j=1}^{N} E_{0j}\hat{\mathbf{z}}\left(e^{i\delta_{j}}e^{i\mathbf{k}_{j}\cdot\mathbf{a}}e^{i\mathbf{k}_{j}\cdot\mathbf{r}}+e^{-i\delta_{j}}e^{-i\mathbf{k}_{j}\cdot\mathbf{a}}e^{-i\mathbf{k}_{j}\cdot\mathbf{r}}\right)$,

We note that *N* can approach infinity and the summation may become an integration in the general case. Accordingly Eq. represents the most general form of TE standing wave. The far field scattered wave for the incident field is given by

$\mathbf{E}_{s}=\sum_{j=1}^{N} E_{0j}\left[e^{i\delta_{j}}e^{i\mathbf{k}_{j}\cdot\mathbf{a}}\mathbf{f}\left(\hat{\mathbf{n}},\mathbf{k}_{j}\right)+e^{-i\delta_{j}}e^{-i\mathbf{k}_{j}\cdot\mathbf{a}}\mathbf{f}\left(\hat{\mathbf{n}},-\mathbf{k}_{j}\right)\right]\frac{e^{ikr}}{r}$.

We shall now prove by mathematical induction that $\mathbf{F}_{recoil}$ is conservative when a spherical particle is illuminated by a TE standing wave Eq. . We shall first prove that $\mathbf{F}_{recoil}$ is conservative for *N* pairs of counter-propagating plane waves satisfying , where *N* is an integer greater than or equal to 1. Then we show that if $\mathbf{F}_{recoil}$ is conservative for *N* pairs of counter-propagating plane waves, it is also true for *N*+1 pairs.

According to the Supplementary Information of Ref. 2, $\mathbf{F}_{\text{recoil}}$ can be expressed as

$$\mathbf{F}_{recoil}=-\frac{1}{c}\oint_{S_{\infty}} \mathbf{S}_{s}d\sigma=-\frac{1}{2c}\oint_{S_{\infty}} \mathrm{Re}[\mathbf{E}_{s}\times\mathbf{H}_{s}^{*}]d\sigma=-\frac{\varepsilon_{0}}{2}\oint_{S_{\infty}} |\mathbf{E}_{s}|^{2}\hat{\mathbf{n}}d\sigma,$$

where

$$\mathbf{S}_{s}=\frac{1}{2}\mathrm{Re}\left\{\mathbf{E}_{s}\times\mathbf{H}_{s}^{*}\right\}$$

is the time averaged Poynting vector of the scattered field, and $S_{\infty}$ is a spherical surface at infinity.

Consider a spherical particle illuminated by a standing wave formed by one single pair of counter-propagating plane waves. By symmetry, $\mathbf{F}_{recoil}$ must be parallel to **k** and is a function of $\mathbf{x}\cdot\mathbf{k}$: $\mathbf{F}_{recoil}=f\left(\mathbf{x}\cdot\mathbf{k}\right)\mathbf{k}$. Clearly, $\nabla\times\mathbf{F}_{recoil}=\mathbf{0}$, indicating $\mathbf{F}_{recoil}$ is conservative for *N* pair of standing wave, where *N* is an integer greater or equal to 1.

We next show that if $\mathbf{F}_{recoil}$ for *N* pairs of standing waves is conservative, where $N\geq1$, it is also true for *N*+1 pairs. Substituting the expression of $\mathbf{E}_{s}$in Eq. into Eq. , and this gives

$\mathbf{F}_{recoil}=\sum_{j=1}^{N+1} \mathbf{h}_{j,-j}+\sum_{j=1}^{N} \sum_{i=1}^{j-1} \mathbf{g}_{i,j}\text{+}\sum_{i=1}^{N} \mathbf{g}_{i,\left(N+1\right)}$.

We note that the first term in is just the force acting on the particle due to each pairs of counter-propagating plane waves, and this is known to be conservative as argued above. The second term in is the cross terms between the first *N* pairs of the plane waves. Since the force induced by the first *N* pairs of waves is conservative, the second term is also conservative. Accordingly, we only need to prove that the third term is conservative, where the third term is given by

$\sum_{i=1}^{N} \mathbf{g}_{i,\left(N+1\right)}=-\varepsilon_{0}E_{0j}E_{0(N+1)}\mathrm{Re}\left[\sum_{i=1}^{N} \oint_{\Omega} \left\{\begin{matrix} +e^{i(\delta_{N+1}+\delta_{i})}e^{i(\mathbf{k}_{N+1}+\mathbf{k}_{i})\cdot\mathbf{a}}\left[\begin{matrix} +\mathbf{f}\left(\hat{\mathbf{n}},\mathbf{k}_{N+1}\right)\cdot\mathbf{f}^{*}\left(\hat{\mathbf{n}},-\mathbf{k}_{i}\right) \\ +\mathbf{f}^{*}\left(\hat{\mathbf{n}},-\mathbf{k}_{N+1}\right)\cdot\mathbf{f}\left(\hat{\mathbf{n}},\mathbf{k}_{i}\right) \end{matrix}\right] \\ +e^{i(\delta_{N+1}-\delta_{i})}e^{i(\mathbf{k}_{N+1}-\mathbf{k}_{i})\cdot\mathbf{a}}\left[\begin{matrix} +\mathbf{f}\left(\hat{\mathbf{n}},\mathbf{k}_{N+1}\right)\cdot\mathbf{f}^{*}\left(\hat{\mathbf{n}},\mathbf{k}_{i}\right)] \\ +\mathbf{f}^{*}\left(\hat{\mathbf{n}},-\mathbf{k}_{N+1}\right)\cdot\mathbf{f}\left(\hat{\mathbf{n}},-\mathbf{k}_{i}\right) \end{matrix}\right] \end{matrix}\right\}\widehat{\mathbf{n}}d\Omega\right]$.

Noting that vector dot product is invariant upon spatial inversion, we have

$\begin{matrix} \oint_{\Omega} \mathbf{f}^{*}\left(\hat{\mathbf{n}},-\mathbf{k}_{N+1}\right)\cdot\mathbf{f}\left(\hat{\mathbf{n}},\mathbf{k}_{i}\right)\hat{\mathbf{n}}d\Omega=\oint_{\Omega} \mathbf{f}^{*}\left(-\hat{\mathbf{n}},\mathbf{k}_{N+1}\right)\cdot\mathbf{f}\left(-\hat{\mathbf{n}},-\mathbf{k}_{i}\right)\hat{\mathbf{n}}d\Omega, \\ =\oint_{\Omega} \mathbf{f}^{*}\left(\hat{\mathbf{n}},\mathbf{k}_{N+1}\right)\cdot\mathbf{f}\left(\hat{\mathbf{n}},-\mathbf{k}_{i}\right)\left(-\hat{\mathbf{n}}\right)d\Omega. \end{matrix}$,

where we change$-\hat{\mathbf{n}}$for $\hat{\mathbf{n}}$ in going from the first line to the second line. Similarly,

$$\begin{matrix} \oint_{\Omega} \mathbf{f}^{*}\left(\hat{\mathbf{n}},-\mathbf{k}_{N+1}\right)\cdot\mathbf{f}\left(\hat{\mathbf{n}},-\mathbf{k}_{i}\right)\hat{\mathbf{n}}d\Omega=\oint_{\Omega} \mathbf{f}^{*}\left(-\hat{\mathbf{n}},\mathbf{k}_{N+1}\right)\cdot\mathbf{f}\left(-\hat{\mathbf{n}},\mathbf{k}_{i}\right)\hat{\mathbf{n}}d\Omega\\ =\oint_{\Omega} \mathbf{f}^{*}\left(\hat{\mathbf{n}},\mathbf{k}_{N+1}\right)\cdot\mathbf{f}\left(\hat{\mathbf{n}},\mathbf{k}_{i}\right)\left(-\hat{\mathbf{n}}\right)d\Omega\end{matrix}$$

Substituting Eq. and into Eq. , one arrives at

where the unit vectors $\hat{\mathbf{x}}_{\left(\mathbf{k}_{N+1},-\mathbf{k}_{i}\right)}$ and alike are for the coordinate system defined by

$$\begin{matrix} \hat{\mathbf{x}}_{\left(\mathbf{k},\mathbf{k}'\right)}=\left(\mathbf{k}-\mathbf{k}'\right)/\left|\mathbf{k}-\mathbf{k}'\right| \\ \hat{\mathbf{y}}_{\left(\mathbf{k},\mathbf{k}'\right)}=\left(\mathbf{k}+\mathbf{k}'\right)/\left|\mathbf{k}+\mathbf{k}'\right| \\ \hat{\mathbf{z}}_{\left(\mathbf{k},\mathbf{k}'\right)}=\hat{\mathbf{x}}_{\left(\mathbf{k},\mathbf{k}'\right)}\times\hat{\mathbf{y}}_{\left(\mathbf{k},\mathbf{k}'\right)}. \end{matrix}$$

and $\theta_{\left(\mathbf{k}_{N+1},-\mathbf{k}_{i}\right)}$and are the spherical coordinates to that coordinate system. With mirror symmetry about the plane spanned by $\hat{\mathbf{x}}_{\left(\mathbf{k}_{N+1},-\mathbf{k}_{i}\right)}$ and $\hat{\mathbf{z}}_{\left(\mathbf{k}_{N+1},-\mathbf{k}_{i}\right)}$axis, which maps $\mathbf{k}_{N+1}\leftrightarrow-\mathbf{k}_{i}$ and $\hat{\mathbf{n}}\leftrightarrow\hat{\mathbf{n}}_{b}$ where , we arrive

$\begin{matrix} \mathbf{f}\left(\hat{\mathbf{n}},\mathbf{k}_{N+1}\right)=\mathbf{f}\left(\hat{\mathbf{n}}_{b},-\mathbf{k}_{i}\right) \\ \mathbf{f}\left(\hat{\mathbf{n}},-\mathbf{k}_{i}\right)=\mathbf{f}\left(\hat{\mathbf{n}}_{b},\mathbf{k}_{N+1}\right) \end{matrix}$.

It then follows that

$$\mathrm{Im}\left\{\mathbf{f}\left(\hat{\mathbf{n}},\mathbf{k}_{N+1}\right)\cdot\mathbf{f}^{*}\left(\hat{\mathbf{n}},-\mathbf{k}_{i}\right)\right\}=\mathrm{Im}\left\{\mathbf{f}\left(\hat{\mathbf{n}}_{b},-\mathbf{k}_{i}\right)\cdot\mathbf{f}^{*}\left(\hat{\mathbf{n}}_{b},\mathbf{k}_{N+1}\right)\right\}=-\mathrm{Im}\left\{\mathbf{f}\left(\hat{\mathbf{n}}_{b},\mathbf{k}_{N+1}\right)\cdot\mathbf{f}^{*}\left(\hat{\mathbf{n}}_{b},-\mathbf{k}_{i}\right)\right\}$$

Accordingly, $\mathrm{Im}\left\{\mathbf{f}\left(\hat{\mathbf{n}},\mathbf{k}_{N+1}\right)\cdot\mathbf{f}^{*}\left(\hat{\mathbf{n}},-\mathbf{k}_{i}\right)\right\}$ is odd in , since implies $\hat{\mathbf{n}}\to\hat{\mathbf{n}}_{b}$. Upon integration of , one arrives at

as these expressions are odd in . Accordingly, the first term in is parallel to $\hat{\mathbf{y}}_{\left(\mathbf{k}_{N+1},-\mathbf{k}_{i}\right)}=\left(\mathbf{k}_{N+1}+\mathbf{k}_{i}\right)/\left|\mathbf{k}_{N+1}+\mathbf{k}_{i}\right|$. Using a similar approach, one can show that the second term is parallel to $\hat{\mathbf{y}}_{\left(-\mathbf{k}_{N+1},-\mathbf{k}_{i}\right)}=\left(-\mathbf{k}_{N+1}+\mathbf{k}_{i}\right)/\left|-\mathbf{k}_{N+1}+\mathbf{k}_{i}\right|$. Consequently, one may write

$\sum_{i=1}^{N} \mathbf{g}_{i,\left(N+1\right)}=-2\varepsilon_{0}E_{0j}E_{0(N+1)}\mathrm{Re}\sum_{i=1}^{N} \oint_{\Omega} \left\{\begin{matrix} +e^{i(\delta_{N+1}+\delta_{i})}e^{i(\mathbf{k}_{N+1}+\mathbf{k}_{i})\cdot\mathbf{a}}\left(\mathbf{k}_{N+1}+\mathbf{k}_{i}\right)\left(...\right) \\ +e^{i(\delta_{N+1}-\delta_{i})}e^{i(\mathbf{k}_{N+1}-\mathbf{k}_{i})\cdot\mathbf{a}}\left(-\mathbf{k}_{N+1}+\mathbf{k}_{i}\right)\left(...\right) \end{matrix}\right\}d\Omega$,

where the quantities inside are independent of $\mathbf{a}$. Clearly

$\nabla\times\sum_{i=1}^{N} \mathbf{g}_{i,\left(N+1\right)}=\mathbf{0}$.

This concludes that $\mathbf{F}_{recoil}$is conservative for a TE standing wave characterized by .

**Conclusion**

The optical force induced by a TE standing wave is conservative. By using a similar approach, one can show that the optical force induced by a TM standing wave is also conservative. For 3D situation, in addition to the standing wave condition, additional symmetries are required. Indeed, we did find some special cases in 3D where the optical force is fully conservative for an A

**A3. The importance of having the correct phase to fulfill the standing wave condition**

The analytical approach in part one of this Supplementary Information showed that the optical force is conservative for a 2D configuration of standing waves. Figure A1(b) below shows the ratio of the gradient force to the scattering force for the configuration shown in Fig. A1(a). It is clear that the phase is important in producing a conservative force.


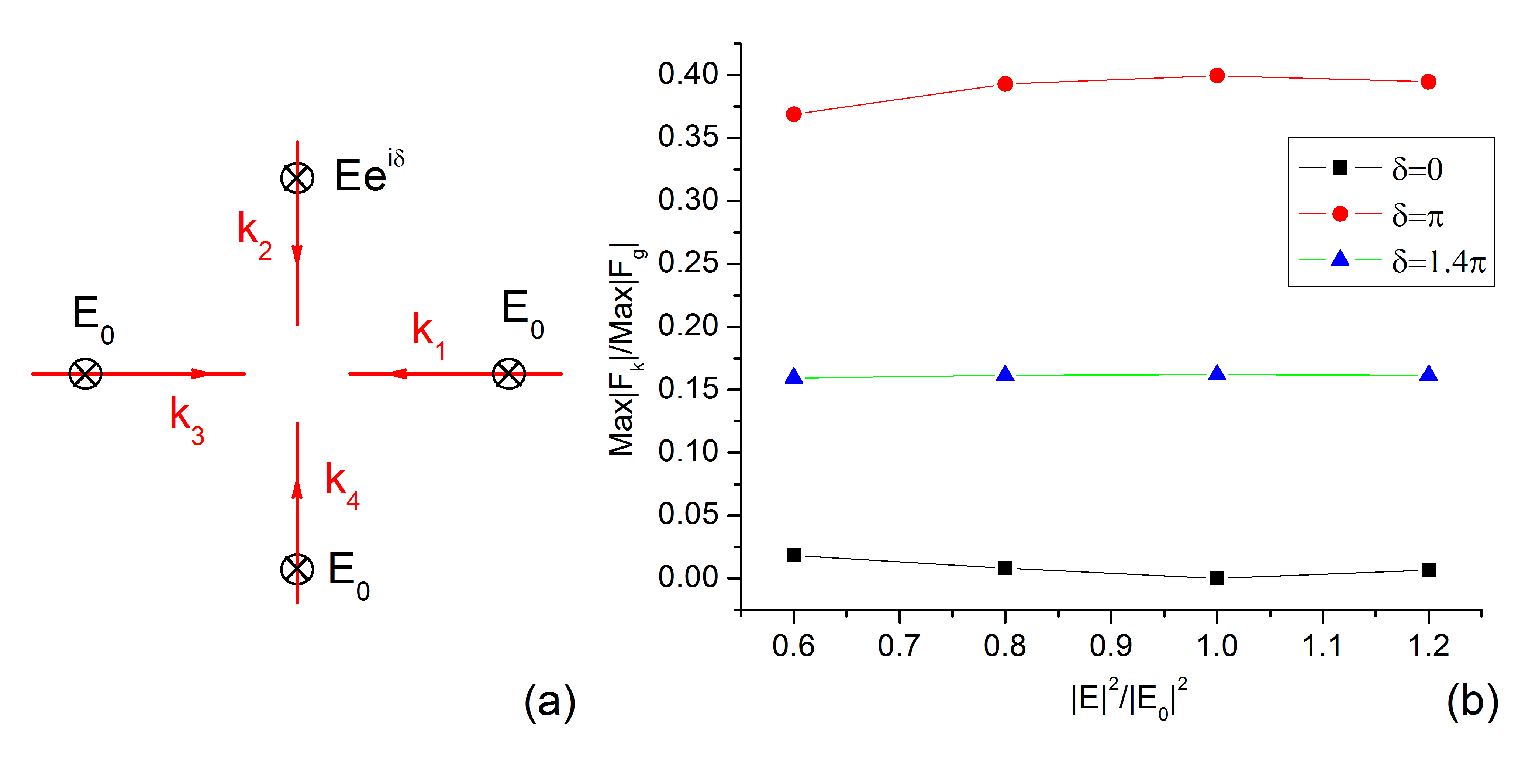


Fig. A1. (a) Schematic of two pairs of counter-propagating plane wave. (b) The ratio of the gradient force to scattering force for the configuration showed in (a). It is clear that this ratio depends strongly on the phase and therefore the standing wave condition is very important.

1. J. D. Jackson, *Classical Electrodynamics* 3^rd^ ed.*,* (Wiley & Sons, 1998). [↑](#endnote-ref-1)
2. Chen, J., Ng, J., Lin, Z. F., and Chan, C. T., Nature Photon. **5**, 531-534 (2011). [↑](#endnote-ref-2)
3. F. Bohren, R. Huffman, Absorption and Scattering of Light by Small Particles, (Wiley, 1983). [↑](#endnote-ref-3)
4. A. Ashkin, J. P. Gordon, Stability of radiation-pressure particle traps: an optical Earnshaw theorem, Opt. Lett. **8**, 511 (1983). [↑](#endnote-ref-4)
5. M. Nieto-Vesperinas, J. J. Saenz, R. Gomez-Medina1 and L.Chantada, Optical forces on small magnetodielectric particles, Opt. Exp. **18**, 11428 (2010). [↑](#endnote-ref-5)
